# Efficacy of interactive manual dexterity training after stroke: a pilot single-blinded randomized controlled trial

**DOI:** 10.1186/s12984-023-01213-9

**Published:** 2023-07-18

**Authors:** Maxime Térémetz, Sonia Hamdoun, Florence Colle, Eloïse Gerardin, Claire Desvilles, Loïc Carment, Sylvain Charron, Macarena Cuenca, David Calvet, Jean-Claude Baron, Guillaume Turc, Marc A. Maier, Charlotte Rosso, Jean-Louis Mas, Påvel G. Lindberg

**Affiliations:** 1 Institute of Psychiatry and Neuroscience of Paris, INSERM U1266, Université Paris Cité, 102-108 Rue de La Santé, 75014 Paris, France; 2 Service de Médecine Physique et de Réadaptation, Groupe Hospitalier Universitaire Paris, Psychiatrie et Neurosciences, 1 Rue Cabanis, 75014 Paris, France; 3 SSR Neurologique, Hôpitaux de Saint-Maurice, 12/14 Rue du Val d’Osne, 94410 Saint-Maurice, France; 4 Neurology Department, Stroke Unit, UCLouvain/CHU UCL Namur (Godinne), Yvoir, Belgium; 5 Centre de Recherche Clinique, Groupe Hospitalier Universitaire Paris, Psychiatrie et Neurosciences, 1 Rue Cabanis, 75014 Paris, France; 6 Service de Neurologie, Groupe Hospitalier Universitaire Paris, Psychiatrie et Neurosciences, 1 Rue Cabanis, 75014 Paris, France; 7 Université Paris Cité, CNRS, Integrative Neuroscience and Cognition Center, 75006 Paris, France; 8 Institut du Cerveau et de la Moelle Épinière, ICM, Inserm U 1127, CNRS UMR 7225, Sorbonne Université, Paris, France; 9 FHU NeuroVasc, Paris, France

**Keywords:** Stroke, RCT, Upper limb, Finger training, Dexterity, Hand use

## Abstract

**Objective:**

To compare the efficacy of Dextrain Manipulandum™ training of dexterity components such as force control and independent finger movements, to dose-matched conventional therapy (CT) post-stroke.

**Methods:**

A prospective, single-blind, pilot randomized clinical trial was conducted. Chronic-phase post-stroke patients with mild-to-moderate dexterity impairment (Box and Block Test (BBT) > 1) received 12 sessions of Dextrain or CT. Blinded measures were obtained before and after training and at 3-months follow-up. Primary outcome was BBT-change (after–before training). Secondary outcomes included changes in motor impairments, activity limitations and dexterity components. Corticospinal excitability and short intracortical inhibition (SICI) were measured using transcranial magnetic stimulation.

**Results:**

BBT-change after training did not differ between the Dextrain (N = 21) vs CT group (N = 21) (median [IQR] = 5[2–7] vs 4[2–7], respectively; P = 0.36). Gains in BBT were maintained at the 3-month post-training follow-up, with a non-significant trend for enhanced BBT-change in the Dextrain group (median [IQR] = 3[− 1–7.0], P = 0.06). Several secondary outcomes showed significantly larger changes in the Dextrain group: finger tracking precision (mean ± SD = 0.3 ± 0.3N vs − 0.1 ± 0.33N; P < 0.0018), independent finger movements (34.7 ± 25.1 ms vs 7.7 ± 18.5 ms, P = 0.02) and maximal finger tapping speed (8.4 ± 7.1 vs 4.5 ± 4.9, P = 0.045). At follow-up, Dextrain group showed significantly greater improvement in Motor Activity Log (median/IQR = 0.7/0.2–0.8 vs 0.2/0.1–0.6, P = 0.05). Across both groups SICI increased in patients with greater BBT-change (Rho = 0.80, P = 0.006). Comparing Dextrain subgroups with maximal grip force higher/lower than median (61.2%), BBT-change was significantly larger in patients with low vs high grip force (7.5 ± 5.6 vs 2.9 ± 2.8; respectively, P = 0.015).

**Conclusions:**

Although immediate improvements in gross dexterity post-stroke did not significantly differ between Dextrain training and CT, our findings suggest that Dextrain enhances recovery of several dexterity components and reported hand-use, particularly when motor impairment is moderate (low initial grip force). Findings need to be confirmed in a larger trial.

*Trial registration* ClinicalTrials.gov NCT03934073 (retrospectively registered)

**Supplementary Information:**

The online version contains supplementary material available at 10.1186/s12984-023-01213-9.

## Introduction

Despite spontaneous recovery with conventional rehabilitation, over half of stroke survivors may retain a disabling motor deficit in the chronic phase, mainly affecting the upper limb [1, 2]. Although impaired manual dexterity and control of the fingers hamper many daily activities, there are currently no specifically targeted treatments for multiple aspects of dexterous manual control. Independent finger movements, a hallmark of manual dexterity in humans [3], is slow to recover after stroke [4]. Recovery of strength and finger individuation partly dissociate during the first 3-months, suggesting separate neural mechanisms driving their recovery [5]. In agreement, our team [6] showed that recovery of finger individuation during the first 6 months post-stroke was slower than that of grip force, and remained significantly impaired despite recovery in corticospinal excitability probed with transcranial magnetic stimulation (TMS). In the chronic phase, impaired strength and finger individuation together best explain impaired dexterous hand use, highlighting that both are essential to recover a functional hand [7]. Other aspects of manual dexterity, such as coordination of finger force in precision grip [8] or the capacity to release grip force abruptly (reflecting motor inhibition) [9], also remain particularly impaired in many chronic stroke survivors and contribute to deficient dexterous hand use.

A recent study reported that training of finger individuation in chronic stroke patients is feasible and can improve finger individuation and lead to lasting improvements in hand function [10]. Training of controlled index finger movements in the chronic post-stroke phase also leads to partially recovered dexterous hand use and is accompanied by reorganization of cortical sensorimotor networks [11]. Friedman et al. [12] showed enhanced recovery of dexterous hand use after MusicGlove training, and piano training may improve motor recovery of individuated finger movements [13]. However, randomized controlled trials are lacking and it remains therefore unclear whether finger-training approaches have enhanced efficacy to improve hand motor impairments and activity limitations compared to conventional therapy.

We have developed dedicated technology to simultaneously measure dexterity components and rehabilitate selective finger movement control [14]. The Dextrain Manipulandum™ allows measurement of flexion–extension finger movements. Finger movement tasks combine visual and auditory feedback, with each task targeting a previously identified specific dexterity component [14]. The four principal exercises include: (i) visuo-motor force-tracking focusing on generation, modulation and inhibition of finger forces; (ii) rhythm tapping for assessing timing of finger movements; (iii) motor sequences for evaluating reproduction and learning of sequential finger movements; and (iv) multi-finger tapping for quantifying finger individuation [6, 14]. This method was shown to be feasible in patients with mild-moderate upper limb impairment, and initial results confirmed differential recovery patterns among dexterity components [6, 14].

The present study aimed to evaluate the “proof-of-concept” benefit of training using the Dextrain Manipulandum for the rehabilitation of hand and fingers in stroke subjects. We hypothesized that training of specific finger dexterity components (e.g., force control and independence of finger movements) coupled with real-time visual feedback and performance scores, enhancing motivation, would lead to greater gains in dexterous hand use compared to dose-matched conventional therapy. A secondary aim was to investigate whether Dextrain therapy leads to greater improvements in hand motor and sensory impairments, quantified dexterity components and activity limitations, as compared to conventional therapy. Finally, to decipher the neurophysiological mechanisms underlying dexterity recovery we explored how motor cortex excitability and short intracortical inhibition (SICI), measured with TMS, changed with therapy and how this change correlated with dexterity improvements.

## Methods

### Study design and participants

This study was reported according to CONSORT guidelines for pilot RCTs [15] (Additional file 1). DEXTRAIN, a pilot randomized, single-blinded trial (NCT03934073), was conducted at the GHU Paris Psychiatrie et Neurosciences hospital, Paris, France from 2018 to 2021. The study consisted of three measurement time-points: before the training (T0), immediately after (T1) and at follow-up 3 months after the end of training (T2). Inclusion criteria included: age > 18 years; patient affiliated to French social security scheme or equivalent; first symptomatic stroke (ischemic or hemorrhagic) dating back 3 months or more; mild-to-moderate gross dexterity impairment, indicated by BBT score < 52 blocks/minute and ≥ 1 block and 10° active extension of the wrist and index metacarpophalangeal joint. Exclusion criteria: multiterritory ischemic stroke; presence of significant disability or pre-existing deficit that could interfere with study treatments/assessments, e.g. severe aphasia, dementia; cognitive impairment, i.e. Mini Mental State Examination (MMSE) score < 25; botulinum toxin treatment of spastic upper limb muscles < 3 months before inclusion and/or planned during rehabilitation protocol; other severe illness making follow-up difficult. None of the patients were receiving other upper limb therapies for the duration of the study. Included stroke patients had the option to participate in the ancillary TMS and functional MRI study. Specific contraindications for MRI and TMS assessments: presence of MR unsafe implants; epilepsy or history of epileptic episode; recreational drug use; excessive alcohol consumption or taking medications that can modify TMS measurements; pregnancy or breastfeeding; participation in another therapeutic study. Ten patients underwent TMS before and after therapy. A group of healthy controls, of comparable age (N = 30, 18 females, age 63.5 ± 10.3 years), without any neurological, orthopedic or other condition affecting hand function, were included to obtain reference values for a subset of motor impairment measures.

The study was conducted according to established good clinical practice guidelines and was approved by an independent ethical committee (CPP Sud-Est I #2017-56). Written informed consent from each participant was obtained.

### Randomization and masking

The Clinical Research Unit, GHU Paris Psychiatrie et Neurosciences hospital administered the randomization through a predetermined centralized web-based randomization system to either Dextrain training or CT in blocks of 4 patients. Randomization was stratified on BBT scores [≥ 1 and ≤ 26 blocks] or [≥ 27 and ≤ 52 blocks] and side of stroke lesion (right/left). All measurements were performed by a trained researcher (MT) blinded to treatment allocation. Two experienced occupational therapists (CD, EG) performed all training sessions.

### Intervention

A single Dextrain training session consisted of 20 min of conventional training (see below) followed by 40 min of Dextrain exercises (www.dextrain.com) targeting the different components of dexterity. This was to ensure that patients received some proximal arm therapy and stretching for spastic muscles as needed [16]. The Dextrain Manipulandum is equipped with 5 force sensors (pistons). Each piston is activated independently by a finger, and flexion and extension forces exerted are recorded and displayed on a screen in real-time. To exert extension movements, small flat magnets were attached to the finger tips with adhesive tape (allowing recording of piston extension forces of up to 0.5N). Spring-loaded pistons have an initial dynamic movement range (1 cm in flexion, 0.5 cm in extension), beyond which isometric forces are measured. Four tasks were developed to assess dexterity components [6, 14] and here these tasks were also used to train dexterity (i.e., patients in both groups were tested at three time-points using Dextrain tasks and the Dextrain group trained on these same tasks). Each task lasts 3–4 min and addresses a specific aspect of finger motor control:

- i. finger force-tracking
  This task probes visuo-motor force control. The subject is required to precisely generate and modulate fingertip forces in one finger to follow a target force trajectory as closely as possible. The subject gets real-time visual feedback on exerted force (cursor) and matches this to the displayed target force (a line passing from right to left on the screen). Each trial consisted of a ramp-phase (linearly increasing force), a hold-phase (stable 2N force) and a release-phase (instantaneous return to the resting force level), followed by a resting-phase. The abrupt release-phase was conceived to measure (and rehabilitate) ability to stop voluntary contractions, an important aspect of force control that explains additional variance of functional grip capacity [9, 17, 18]. A force-tracking session consisted of 48 trials (8 blocks of 6 trials). The target forces correspond to force range employed in daily object manipulations [19]. Patients performed tracking mostly with the thumb, index or middle finger of the paretic hand. Overall task performance feedback was indicated at the end of a session by displaying mean force tracking error (N) and release duration (ms).
- ii. finger motor sequences
  This task was designed to test the capacity to learn a simple finger-tapping motor sequence [14]. The subject is presented with a (visual) five-finger tapping sequence, randomly selected from 3 different pseudo-randomized sequences. A given sequence is first practiced ten times with visual feedback. Then the subject is required to repeat the sequence from memory (and without feedback). There is no instruction regarding force amplitude of finger taps. Overall task performance feedback was provided at the end of a session by displaying success rate (%).
- iii. rhythm tapping
  This task assesses the ability to perform and maintain repetitive finger tapping at frequencies indicated by auditory cues. The subject is instructed to tap, with a single finger, in time (simultaneously) with an auditory cue (beep). Each of the five fingers is tested at three frequencies (1, 2 or 3 Hz). A first period of 30 taps with auditory cue is followed by a period of 30 taps without cue. Previous work showed that stroke patients have greatest difficulty to achieve 3 Hz tapping [6, 14]. Overall task performance feedback was given at the end of a session by displaying the mean and SD of the tapping frequency (Hz) and the variability of the intertap interval (ms).
- iv. independence of finger movements
  This task assesses the ability to perform independent finger movements which are characterized by isolated movement execution (selection) of one finger, while inhibiting movements of neighboring fingers not involved in the trial [6, 14]. The trials in a session included one-finger and two-finger taps. A typical session included 90 trials, each lasting 3 s. The configurations varied trial-by-trial (pseudo-randomized) and consisted of one-finger taps (separate tap of index, middle, ring or little finger) and two-finger taps (simultaneous index-middle, index-ring, index-little, middle-ring, middle-little or ring-little finger taps). The target (tap) finger(s) was visually cued, and real-time feedback provided for each tap. Subjects were instructed to execute the tap following the cue as fast as possible, and to prioritize correct finger selection (over force of taps). Overall task performance feedback was provided at the end of a session by displaying success rate (%), co-activation (in %), and reaction times (ms) to single and two-finger combinations separately.

#### Adapting the Dextrain therapy to the individual

Therapists were instructed to prioritize finger force tracking and multi-finger tapping tasks for training, as both have been shown to improve with training [20]. Within the 40 min time window, the therapist was free to choose how many of each task to train, and also to combine with the other two tasks (rhythm tapping and finger motor sequences). The difficulty level of each exercise was adapted, across the 12 training sessions, according to the degree of impairment of each patient. Severely impaired patients performed more force control exercises (feasible for all patients). More difficult tasks (rhythm, sequences and independence of finger movements) were successively introduced during the training sessions as patients improved their ability to maintain the fingers on the pistons and to achieve the tasks. Overall mean performance results were shown at the end of each task.

Conventional therapy (CT) consisted of 60 min of occupational hand and dexterity training. The exercises used in each therapy session, for each patient, were recorded according to OT-star [21] and included: mobilizing muscles and soft tissues, strengthening exercises, facilitation of movements, sensory exercises (proprioception, touch, texture, and stereognosis), training of dexterity and fine motor skills, grasp and release, reach and grasp, push and pull, bilateral exercises, and practice of functional upper limb tasks (e.g., polishing, hand washing). CT included visual feedback of active and passive movements (patients had eyes open) but did not provide specific visual feedback of ongoing finger movements.

All patients, regardless of the rehabilitation group thus underwent rehabilitation adapted to the severity of their hand and finger motor impairment in order to optimize training.

### Outcome measures

All outcomes were obtained before training, at the end of training and at 3 months follow-up. The* primary outcome* was treatment-induced change, after the 4-week training program (T1-T0) and at follow-up (T2-T0), in the Box and Block Test score (BBT). The BBT assesses gross dexterous hand use (grip/displace wooden cubes) [22].

#### Secondary outcomes

Maximal grip force (% strength of paretic vs non-paretic hand) to assess strength deficits (Jamar dynamometer; https://www.kinetec-byvivadia.com) [23].

Moberg pick-up test (MPUT) score was used as a functional assessment of precision grip in the paretic hand, i.e. time taken to pick-up and place 12 objects into the box [24].

Light touch sensation was assessed using Semmes–Weinstein monofilaments (Touch Test Sensory Evaluators, 5 item-kit, North Coast Medical; five calibers from 0.07 to 279 g) to measure the tactile sensitivity of finger tips of the paretic hand.

Maximal index-thumb tapping speed, reflecting degree of motor impairment, was assessed by counting the number of index-thumb taps in a period of 15 seconds [25].

Motor Activity Log (MAL), a self-reported, semi-structured questionnaire, was used to evaluate real-life paretic arm and hand use in a range of daily tasks. It separately assesses how much (amount of use) and how well (quality of movement) patients use their paretic arm [26].

Arm Research Action Test (ARAT), a clinical test for grasp, grip, pinch and gross movement in the hemiparetic hand, was used as a global measure of hand function [27].

Dexterity components, measured using the same Dextrain Manipulandum tasks as in training, included the following variables from each of the four tasks:

1. finger force-tracking: Tracking error reflecting the accuracy of force control [28, 29]. Good accuracy is represented by low tracking error, calculated as the root-mean-square error (RMSE) between the applied force and the target force during the ramp-and-hold trajectory. We also measured the ability to quickly release force (release duration = RD) at the end of each ramp-and-hold trial. Release duration was computed as the time taken to reduce the force from 75 to 25% of the target force. Tracking error and release duration have been shown to be increased after stroke [9].
2. rhythm tapping: average tapping rate at 3 Hz pacing. Separate rate (and variability) across trials with and trials without auditory cues.
3. finger motor sequences: few patients were able to perform this task at T0. It was not analyzed at group level.
4. independence of finger movements: Success rate, reflecting the % correct single- or two-finger taps. We also measured the finger tap reaction time [14].

### Transcranial magnetic stimulation (TMS)

Neuronavigated TMS was used to measure motor evoked potential (MEPs; see Additional file 2). Resting motor threshold (rMT) and short-latency intracortical inhibition was measured (SICI; conditioning stimulus at 80%rMT 2 ms prior the test pulse at 120%rMT) [30]. SICI was measured as the % reduction of the conditioned/unconditioned MEP. SICI has been shown to be reduced in acute stroke and may normalize during recovery from motor impairment [31].

### Sample size calculation

A power calculation was based on reported BBT-change after CT [32], i.e., difference ± SD = 4.3 ± 5.4 blocks. We predicted a 50% improvement with Dextrain training (BBT-change of 6.2), since finger training increased the BBT-score by 30% compared to 14% in CT [12]. A sample size = 17 in each group was indicated to obtain power = 80% and alpha risk = 0.05 according to expected BBT-change of 2.1 ± 2.1 between groups. We added 25% to compensate for potential drop-outs (sample size = 21 for each group).

### Statistical analysis

Categorical variables were described as number (%). Data distributions of continuous variables were analyzed using skewness (< − 1 or > 1 = highly skewed data) and Shapiro–Wilk’s test. Normally distributed variables were described as means ± SD and compared with Student t-test, whereas non-normally distributed variables were described as medians (interquartile range [IQR]) and compared with non-parametric tests (Mood median test or Mann–Whitney U test).

The effect of training was evaluated using change variables T1-T0 (absolute difference, reflecting immediate training effect) and T2-T0 (3-month follow-up effect), as specified in the statistical analysis protocol established before project start. The Hodges-Lehmann estimator of median of group differences with its 95% ‘exact’ confidence limits (95%CL) was computed as main measure of treatment effect. To limit the number of statistical tests with regards to secondary outcomes, P-values were only calculated for outcomes where the 95%CL of the median of group differences did not include zero. In this case, the Mood median test was used. Six tests were carried out and we did not correct for multiple comparisons given the exploratory nature of this pilot RCT. Given the group difference in age we also analyzed the percent change in BBT using gender and age normalized BBT scores [22]. The number of patients showing clinically significant change in BBT was reported. As a posthoc, supplemental analysis using parametric statistics to analyze BBT change across time points (T0, T1, T2) was performed using linear mixed models under SPSS. We analyzed (i) BBT scores at all 3 time points including age as a fixed effect covariate and subject as random effect (Additional file 2: Table S2), (ii) age and gender normalized BBT scores across all time points (Additional file 2: Table S3), and (iii) normalized BBT scores with imputation of missing data from T2 (Additional file 2: Table S4). Within-treatment group changes over time were analyzed using Friedman’s repeated measures ANOVA and Wilcoxon’s matched Pairs test. Potential effect of age on treatment effect was assessed using mixed-effects general linear model on time-points (T0, T1, T2) and including age as covariate, after checking that the underlying assumptions were met. Pearson correlation test was used for parametric, and Spearman Rho for non-normal correlation testing.

Finally, to explore the impact of degree of motor impairment on Dextrain training effect we divided patients into moderate and mild motor impairment subgroups using median split of Fmax% and compared BBT-change using Mann–Whitney U Test. Maximal grip force is considered a good overall indicator of upper limb motor impairment [22, 33], related to corticospinal tract excitability [34], even though more recent studies show that maximal grip force is partially dissociated from dexterity recovery [5–7] Statistical analyses were performed using IBM SPSS v23 (www.ibm.com/spss) and SAS 9.4. Statistical significance was set to P ≤ 0.05. No correction for multiple testing was conducted.

## Results

**Fig. 1:**
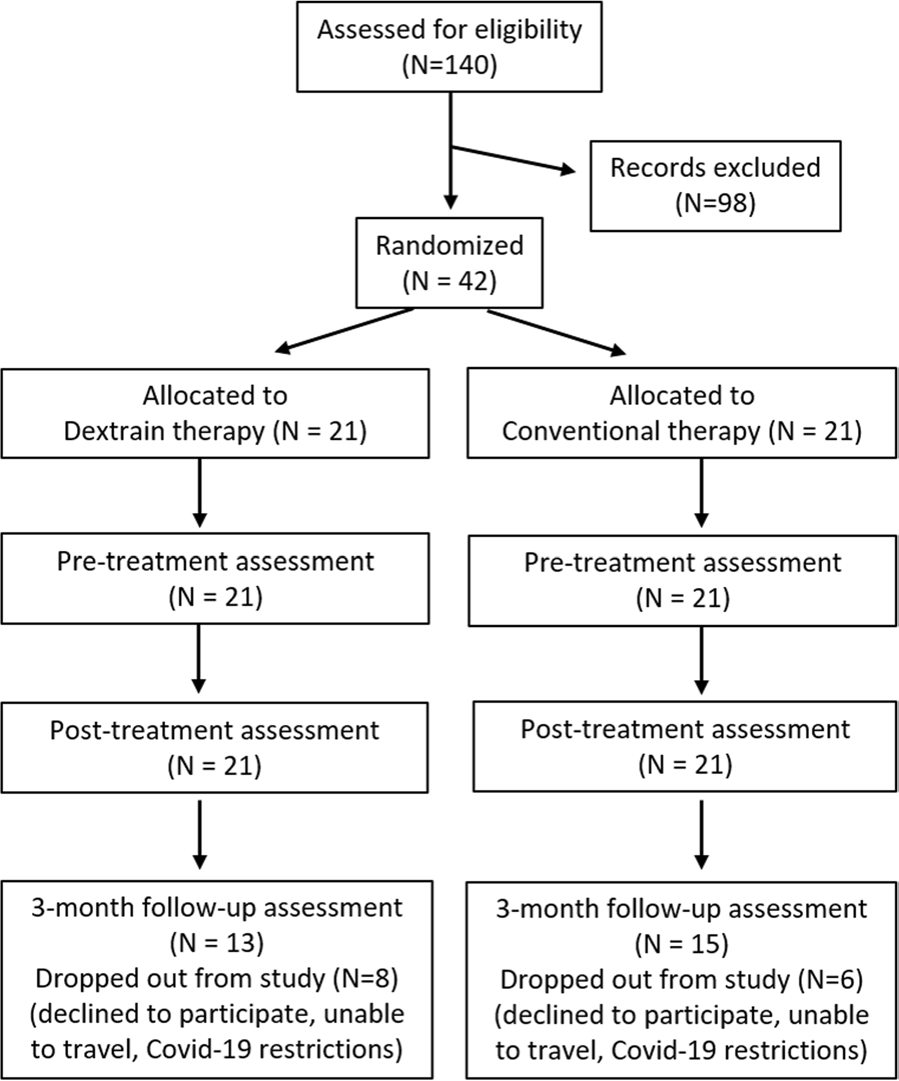
Participant flow through the longitudinal post-stroke study (CONSORT flow chart)

Among 140 stroke patients assessed for eligibility, 42 were included (Fig. 1). All patients completed 12 training sessions except two that completed 11 sessions. Dropout rates at T2 were high in both groups (Fig. 1), largely due to COVID-19 restrictions. Table 1 shows baseline characteristics of each group. Baseline characteristics in age, time since stroke, motor and cognitive impairment were similar in dropouts and non-dropouts (Mann–Whitney U Test, P > 0.2; Additional file 2: Table S5). Time since stroke was similar in both groups (~ 2 years), as was the moderate gross dexterity impairment with BBT score (~ 28 blocks/min). Groups were also similar in other cognitive and motor impairments and activity limitation measures. Dextrain group were 12 years younger on average than the CT group. Therapy sessions consisted of about 60% finger force-tracking, 30% independent finger movements, 5% rhythm tapping and 3% finger motor sequences. No difference between therapists was found in type of Dextrain tasks trained across sessions (Additional file 2: Figure S1).

**Table 1:** Clinical characteristics of the 42 included stroke patients

	Dextrain (N = 21)	CT (N = 21)	P-value (MWU test)
Gender (Male/Female)	16 M, 5 F	13 M, 8 F	
Ischemic/Haemorrhagic stroke	17 I, 4 H	20 I, 1 H	
Paretic side (Right, Left)	13 R, 8 L	12 R, 9 L	
Age (mean ± SD)	58 ± 12	70 ± 16	0.008*
Time since stroke (months, mean ± SD)	23 ± 26	25 ± 27	0.86
MMSE (mean ± SD)	29 ± 1	28 ± 2	0.10
Barthel index (mean ± SD)	97 ± 6	84 ± 25	0.14
BBT (blocks/min) (median, quartile range)	30, 19	28, 24	0.33
ForceMax % (median, quartile range)	63, 13	49, 34	0.11
MPUT (time/object) (median, quartile range)	3, 4	3, 5	0.34*
Max Tapping speed (number of taps in 15 s) (mean ± SD)	32.4 ± 12.0	30.1 ± 11.2	0.47
ARAT (max 57) (median, quartile range)	53, 6	54, 21	0.18

*Student t-test; MWU test: Mann–Whitney U test

Range of clinical scale and clinical cut off value are as follows: MMSE [0–30, ≥ 25 normal], Barthel index [0–100, 100 normal], BBT [0-no upper limit, > 54 blocks/s normal], MPUT [0-60 s per item, ≤ 1.6 normal], ARAT [0–57, 57 normal]. We checked how many patients were in the 3–6 months post-stroke phase, since residual improvement may be greater than after 6 months [35]. Three (of 21) patients in the Dextrain group were 3–6 months post-stroke and 5 (of 21) in the CT group

### Primary outcome

**Fig. 2:**
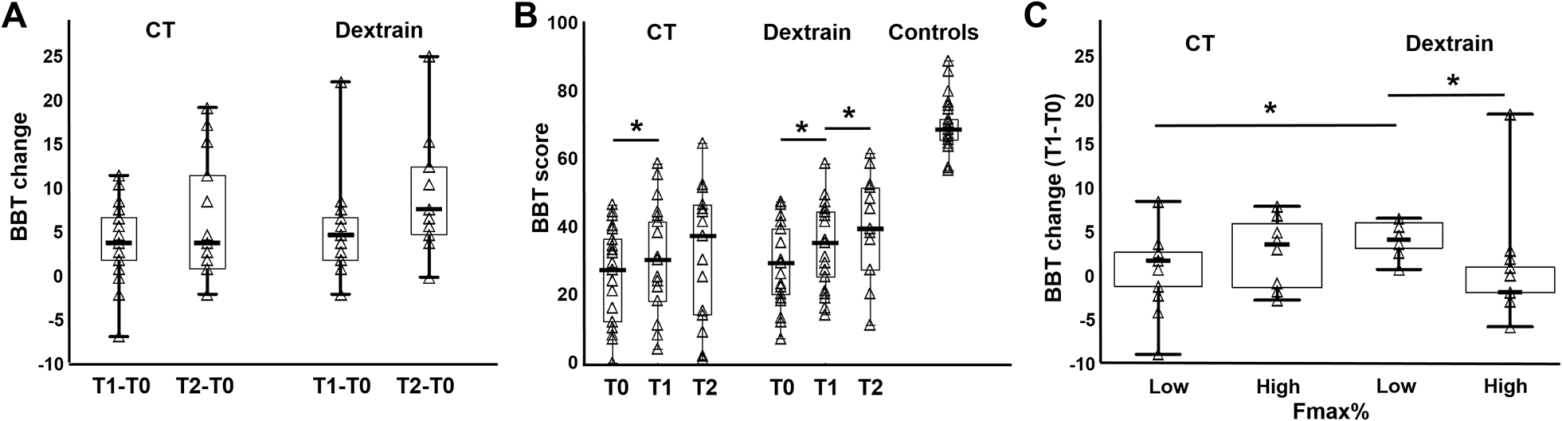
Post-stroke recovery of dexterous hand use. Box and block test (BBT) score as outcome measure across time (training) and groups (Dextrain group vs. CT, conventional therapy group). **A** Primary outcome, BBT-change at post-training (T1-T0), did not differ between Dextrain and CT groups. BBT-change at 3-months follow-up (T2-T0) also did not differ between groups. **B** Within-group BBT scores compared to reference values in healthy control subjects (N = 30) at T0 (baseline), T1 (post-training) and T2 (follow-up); Dextrain and CT groups significantly improved BBT score with training (T1); the CT group showed retention of BBT gains at T2, while the Dextrain group showed further improvement at T2. *P < 0.05. **C** Degree of initial motor impairment and therapy-mediated gains in dexterous hand use. Exploratory analysis after median-splitting the groups according to Fmax% on T0 (line = median, box = 25–75%, whisker = min/max). Dextrain training was more beneficial in patients with moderate motor impairment (Low subgroup) than those with mild motor impairment (High maximal force subgroup; Z = − 2.32, P = 0.02). There was no significant difference in BBT-change between Low and High subgroups within the CT group. The Low motor impairment group also improved more in BBT-change after Dextrain than conventional therapy (Mann–Whitney U Test, P < 0.05)

BBT-change (T1-T0) was not normally-distributed and substantially skewed (Shapiro Wilks = 0.92, P = 0.0045; skewness = 1.06). In the Dextrain group, median (IQR) (T1-T0) BBT-change was 5 (2–7) compared to 4 (2–7) blocks/min in the CT group (Fig. 2A, Table 2), and the median of differences was not significantly different (0.0 [− 3.0 to 2.0], P = 0.36, Table 2). After adjustment for age, using age and gender normalized BBT score change, the median group difference in BBT change (T2-T0) was significantly greater in Dextrain compared to CT (Additional file 2: Figure S2 and Table S1). Nine (43%) patients in the Dextrain group showed a clinically significant change (BBT-change > 5.5 [36]) vs 8 (38%) in the CT group, a similar proportion in each group (*X*^2^ (1, 42) = 1.6, P = 0.81). Gains in BBT were maintained at the 3-month post-training follow-up (T2, Fig. 2A), with a non-significant trend for enhanced BBT-change in the Dextrain group (median of differences: 3.0 [− 1.0 to 7.0], P = 0.06, Table 2). The supplemental posthoc linear mixed models showed no significant interaction of treatment group and time when analyzing raw BBT scores or age and gender normalized BBT scores (Additional file 2: Tables S2-S4).

**Table 2:** Primary and secondary outcomes

	Conventional therapy	Dextrain therapy	Median of differences [CL95%]^†^	P value (Mood median test)
**Clinical assessments**				
**BBT score** (Primary outcome) (median [Q1 to Q3])				
Change from baseline (T0)				
At post-training (T1)	4 [2 to 7]	5 [2 to 7]	0.0 [− 3.0 to 2.0]	0.36
At follow-up (T2)	4 [1 to 12]	8 [5 to 13]	3.0 [− 1.0 to 7.0]	0.06
**Fmax% **(median [Q1 to Q3])				
Change from baseline (T0)				
At post-training (T1)	− 0.7 [− 5.5 to 5.7]	4.2 [− 5.7 to 9.2]	1.9 [− 4.6 to 9.4]	
At follow-up (T2)	3.1 [− 2.1 to 9.6]	8.8 [− 1.8 to 28]	9.9 [− 7.7 to 20.1]	
**MPUT time/item **(median [Q1 to Q3])				
Change from baseline (T0)				
At post-training (T1)	0.5 [0.3 to 1.5]	1 [0.4 to 2.9]	0.3 [− 0.4 to 1.7]	
At follow-up (T2)	0.7 [0.3 to 1.5]	1.2 [0.3 to 3.1]	0.1 [− 0.6 to 2.0]	
**Light touch** (median [Q1 to Q3])				
Change from baseline (T0)				
At post-training (T1)	0 [0 to 3]	0 [0 to 5]	0.0 [0.0 to 3.0]	0.53
At follow-up (T2)	0 [0 to 5]	5 [0 to 8]	3 [0.0 to 5.0]	0.14
**Max Tapping speed **(mean [CI95%])				
Change from baseline (T0)				
At post-training (T1)	4.5 [2.3 to 6.7]	8.4 [5.2 to 11.6]	3.9 [0.0 to 7.7]	0.045*
At follow-up (T2)	7.3 [2.5 to 12.1]	10.6 [3.3 to 17.9]	3.3 [− 5.6 to 12.1]	
**MAL AOU** (median [Q1 to Q3])				
Change from baseline (T0)				
At post-training (T1)	0.3 [0.0 to 0. 7]	0.3 [0.1 to 0.7]	0.1 [− 0.2 to 0.3]	
At follow-up (T2)	0.2 [0.1 to 0.6]	0.7 [0.2 to 0.8]	0.5 [0.0 to 0.7]	0.05*
**MAL QOM** (median [Q1 to Q3])				
Change from baseline (T0)				
At post-training (T1)	0.1 [0.0 to 0.5]	0.3 [0.1 to 0.6]	0.1 [− 0.1 to 0.4]	
At follow-up (T2)	0.3 [0.0 to 0.6]	0.7 [0.2 to 0.8]	0.2 [− 0.1 to 0.7]	
**ARAT **(median [Q1 to Q3])				
Change from baseline (T0)				
At post-training (T1)	2 [0 to 4]	3 [1 to 5]	0.0 [− 1.0 to 2.0]	
At follow-up (T2)	2 [0 to 4]	3 [0 to 7]	0.0 [− 2.0 to 5.0]	
**Dextrain components**				
**Tracking Error **(mean [CI95%])				
Change from baseline (T0)				
At post-training (T1)	− 0.1 [− 0.2 to 0.1]	0.3 [0.2 to 0.4]	0.4 [0.1 to 0.6]	0.0008*
At follow-up (T2)	0.0 [− 0.1 to 0.2]	0.2 [0.0 to 0.4]	0.2 [0.0 to 0.4]	0.06
**Multi-finger Tapping **(independence of finger movements)				
**Success rate** (mean [CI95%])				
Change from baseline (T0)				
At post-training (T1)	0.0 [0.0 to − 0.1]	0.1 [0.0 to 0.2]	0.1 [0.0 to 0.2]	0.27
At follow-up (T2)	0.1 [0.0 to 0.2]	0.1 [− 0.1 to 0.3]	0.0 [− 0.2 to 0.3]	
**Reaction Time** (mean [CI95%])				
Change from baseline (T0)				
At post-training (T1)	7.7 [− 9.5 to 24.8]	34.7 [20.2 to 49.2]	29.9 [15 to 44.9]	0.02*
At follow-up (T2)	2.1 [− 26.8 to 31.1]	30.1 [6.1 to 55.1]	27.5 [4.8 to 50.3]	0.08
**Rhythm 3 Hz** (mean [CI95%])				
Change from baseline (T0)				
At post-training (T1)	0.1 [− 9.9 to 10]	0.4 [− 3.6 to 4.5]	0.4 [− 10 to 10.8]	
At follow-up (T2)	− 0.8 [− 16.4 to 14.8]	− 4.5 [− 13.1 to 4.1]	− 3.8 [− 22.2 to 14.6]	

Mood median test (two-tailed) for variables not normally distributed (Shapiro–Wilk’s test P < 0.05) and Student t-test was used for testing group differences of normally distributed variables. MAL [0–5, 5 normal]. ^†^Hodges–Lehmann estimator of median of differences and ‘exact’ 95% confidence limits (CL)

Light touch sensation (Semmes–Weinstein) [0–10] (0 = not identified, 10 = all correctly identified)

Change from baseline for Tracking error and Reaction Time were calculated as T0-T1 and T0-T2 for post-therapy and follow-up changes, respectively

Note: the median of group differences here shown is in skewed data not (necessarily) identical to the difference of the group medians. P-values calculated when confidence limits of median of differences were positive (not below zero). We did not correct for multiple comparisons since this was a pilot RCT study. Significant differences indicated by *(P ≤ 0.05), provided for median differences with CI in positive range. Fmax% = maximal grip force (%); MPUT = Moberg Pick-up Test; MAL AOU = Motor Activity Log Amount Of Use; MAL QOM = Motor Activity Log Quality Of Movement; ARAT = Action Research Arm Test

Regarding within-group changes, Friedman’s repeated measures ANOVA showed a significant effect of time in both groups (P < 0.001). Wilcoxon matched pairs test showed a significant change immediately post-training (T1-T0) in both groups (Dextrain: Z = 3.8, CT: Z = 3.2, P < 0.002), and a continued positive change after therapy to 3 month follow-up (T2-T1) in the Dextrain (Fig. 2B, Z = 2.55, P = 0.01) but not in the CT group (Z = 0.35, P = 0.73).

Dropouts showed significantly reduced BBT-change (T1-T0) (Additional file 2: Figure S3 and Table S5).

### Secondary outcomes

Secondary outcomes for motor impairment (maximal grip force), sensory impairment (light touch) and activity limitations (MPUT, ARAT, MAL-AOU and MAL-QOM) did not differ between groups immediately after training (Table 2). At 3-month follow-up, the Dextrain group showed a greater median of difference in MAL-AOU than CT (median of differences: 0.5 [0.0 to 0.7], P = 0.05, Table 2). The other measures showed no change.

Regarding within-group changes, Wilcoxon Matched Pairs tests showed that maximal grip force and light touch improved significantly within the Dextrain group post-training (T1) and were maintained at 3 months (T0 *vs*. T2, P < 0.05). In comparison, the changes within the CT group in maximal grip force and light touch were not significant at post-training or at 3 months (T2). Neither Dextrain nor CT groups showed significant improvement in MPUT post-training, but MPUT improvement was significant within both groups at follow-up (T0 vs T2, P < 0.05). Both training groups improved significantly in MAL-AOU, MAL-QOM and ARAT post-training (T1) and ARAT changes remained significant at follow-up. Only the Dextrain group showed significant change (within-group improvement) at 3-month follow-up (T2-T0) in MAL-AOU (Dextrain: Z = 2.55, P = 0.01; CT: Z = 1.57, P = 0.12). Dropouts showed less improvement in maximal tapping rate (Additional file 2: Table S5).

### Manual dexterity components

**Fig. 3:**
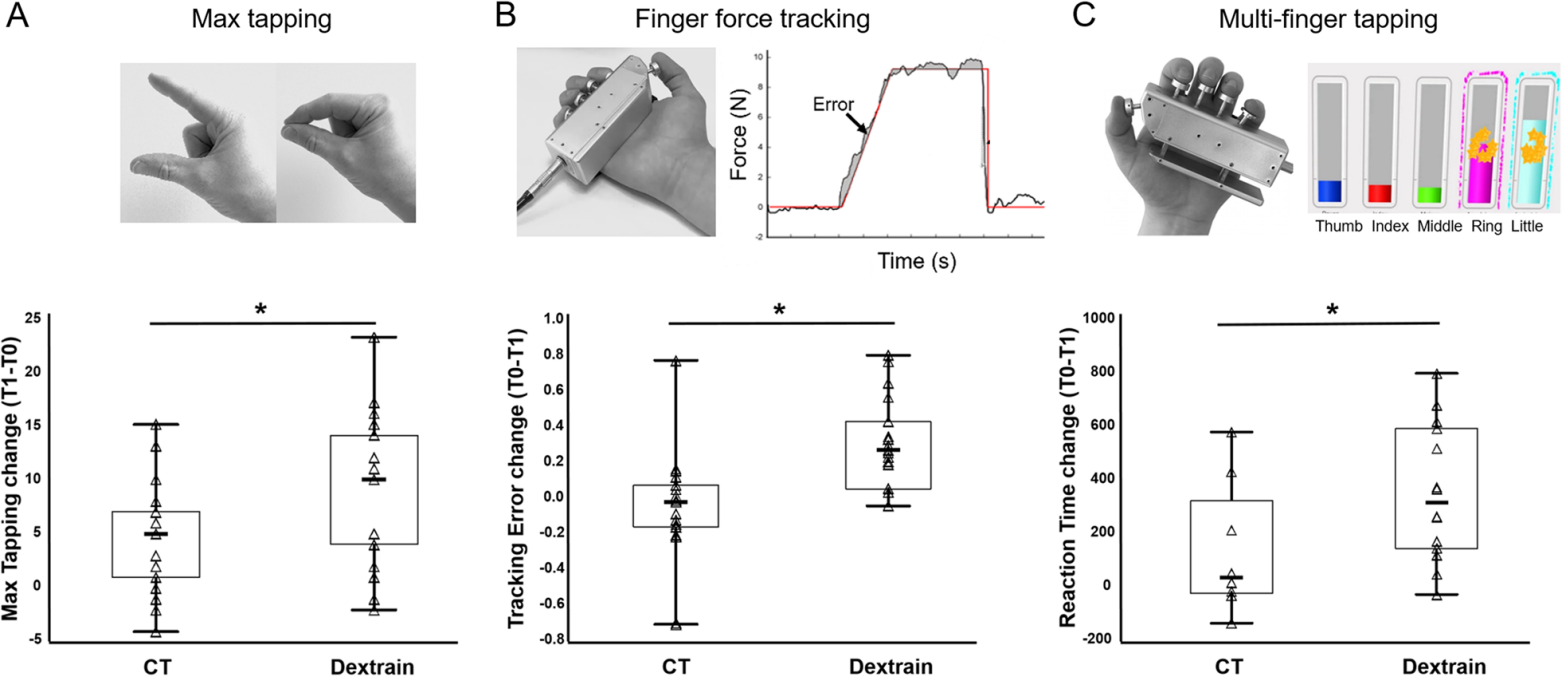
Training effects on components of manual dexterity. **A** Box plots showing mean (line), CI95% (box) and min–max (whiskers) of change scores in dexterity components. Change in maximal tapping speed was significantly greater in Dextrain compared to CT group. **B** Finger force tracking error also improved more in Dextrain compared to CT group. **C** Reaction time change: faster reaction times after training in the Dextrain group compared to CT group (positive change indicates faster times). *P < 0.05 (Student t-test)

Components of manual dexterity showed enhanced improvement in the Dextrain group, including greater improvement in maximal finger tapping speed (Fig. 3A). In the force-tracking task, the Dextrain group showed greater improvement in precision (indicated by reduced error; Fig. 3B) compared to the CT group. In the independent finger movement task, no difference was found for success rate, but the Dextrain group showed a greater reduction in reaction times of selective finger movements (Fig. 3C). Change (T1-T0, i.e., positive values reflect improved performance) in these variables was significantly greater in the Dextrain compared to the CT group (Table 2). The force-tracking error and independent finger movement reaction times both showed non-significant trends (P < 0.1) for a greater change (less error, higher independence) at 3-month follow-up in the Dextrain compared to the CT group (Table 2).

Within the Dextrain group, changes at follow-up (95%CI range at T2-T0, Table 2) in both force-tracking error and independent finger movement reaction times were positive, indicating sustained improvement. In contrast, the 95%CI range of change scores in the CT group at follow-up included negative values, showing that positive change was not sustained in all patients in this group.

No group differences in 3 Hz frequency change were found in the rhythm task.

### M1 excitability and inhibition changes, and relation to BBT-change

**Fig. 4:**
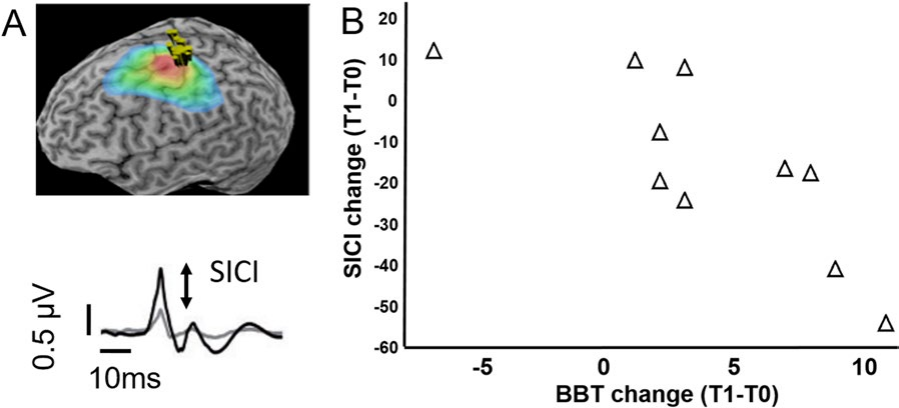
Transcranial magnetic stimulation (TMS) results. **A** Example of the neuronavigation map obtained in a subject during recruitment curve measures. Heat map indicates consistent cortical location of successive stimulations during testing in one patient. This ensured stable cortical location of stimulation over left M1 (red area). Below, an example motor evoked potential in 1DI after single-pulse TMS (in black) and after paired-pulse (conditioned) stimulation (in grey). The difference in MEP size between single and paired pulse stimulations was used to calculate SICI%. **B** Change in SICI% after training (negative values depict reinforced inhibition). The majority of patients showed stronger SICI after training, and the degree of SICI% change correlated significantly with change in BBT (T1-T0; Spearman Rho = − 0.80, P = 0.006)

Combined TMS results across both groups are presented since sample sizes were small (Dextrain N = 3; CT N = 7). Neuronavigated single-pulse TMS (Fig. 4A) in N = 10 patients showed high resting motor thresholds (rMT) in both groups at baseline (Dextrain mean ± SD = 54 ± 13%; CT = 62 ± 10%). No change in motor excitability, indicated by rMT, was found after therapy (change rMT across groups = 2 ± 6%).

A non-significant increment in SICI% (increased inhibition) was found after training (across groups: pre-training = 23 ± 23% vs post-training = 38 ± 26%; P = 0.2).

The relation between change in SICI% and rMT% with BBT-change was studied using Spearman rank correlation. Across the two groups, BBT-change correlated significantly with SICI% change (Rho = − 0.8, P = 0.0056; Fig. 4B), i.e., patients with largest increase in BBT after therapy (T1) showed greatest increase in cortical inhibition. No significant correlation was found between change in rMT% and difference in BBT (Rho = 0.50, P = 0.14).

### Effect of baseline motor impairment on therapy-mediated improvements

To investigate whether BBT change was affected by initial scores (e.g., through ceiling effect) we checked whether patients with high initial BBT scores showed less improvement with training. Patients with high BBT at T0 (> 40) had similar change as patients with lower initial BBT scores, and there was no association between initial BBT scores (T0) and BBT-change (T1-T0) (Additional file 2: Figure S4).

BBT-change was compared after splitting groups according to baseline median Fmax% score = 61.2%, i.e., Fmax% > 61.2% was considered a mild, and Fmax% < 61.2% a moderate motor impairment. BBT-change was compared using Mann–Whitney U test across subgroups. Within the Dextrain group (N = 21), the low Fmax% subgroup had median BBT-change (T1-T0) of 6 points, compared to only 2 points in the high subgroup, which was significantly different (Fig. 2C). BBT change at follow-up (T2-T0) did not show this effect (Z = − 0.54, P = 0.59) but this subgroup analysis was limited to N = 9 with High Fmax% and N = 4 with Low Fmax%.

## Discussion

This randomized, dose-matched, controlled trial did not show a significantly larger effect on BBT-change immediately after the 12 Dextrain therapy sessions compared to conventional post-stroke therapy, with both groups improving similarly, displacing about 5 more blocks/min. About 40% of patients showed a clinically significant BBT difference, indicative of comparable improvement in gross dexterity. Interestingly, however, there was a nearly significant larger improvement in BBT at the 3-month follow-up assessment in the Dextrain group (this difference was significant when using age normalized BBT scores). Although many secondary outcomes also showed similar changes post-training, we found signals for greater improvements in several trained and non-trained dexterity components in the Dextrain group. TMS showed enhanced cortical inhibition correlating with positive BBT-change. An exploratory analysis suggested a greater benefit of Dextrain therapy in patients with moderate (rather than mild) baseline motor impairment.

### Improvements in hand motor impairments and activity limitations

The magnitude of BBT-change found in our chronic patient sample was similar to the average BBT-change of 4.6 blocks/min reported after 120 sessions of functional electrical stimulation training in chronic stroke [37]. A slightly lower BBT-change of 3.2 blocks/min was reported after 6 1-h sessions of MusicGlove training [12]. A study on Wii-therapy including post-stroke patients with severe motor impairment reported a lower effect (0.2 block/min change) after 21 therapy sessions [38]. The disparities in BBT-change in these studies are likely due to differences in hand and finger training intensity and degree of baseline motor impairment [39]. Ideally, movements should be repeated hundreds of times during therapy [40] since intense finger movement training can reduce upper limb impairment in chronic stroke patients [11]. Interactive gaming, as in Dextrain, provides enhanced sensory feedback which may help to increase intensity of rehabilitation [41]. We found no relation between baseline BBT and BBT change (T1-T0; Additional file 2: Figure S4), suggesting that improvements after both CT and Dextrain were not proportional to initial BBT [42]. Perhaps the therapy (either CT or Dextrain) was sufficiently intense to reopen the neuroplasticity window [43].

In addition to BBT improvements, both Dextrain and CT approaches led to similar post-training gains in gross upper limb motor impairment (Fmax), in proximal upper limb movements, grasping and mass finger flexion/extension movements (ARAT), and in self-reported paretic hand use in daily life (MAL). This suggests that Dextrain training improves mass flexion/extension of fingers and control of more proximal arm movements just as conventional therapy does. Transfer from distally trained to proximal arm movements has also been reported after forearm pronation/supination robotic training [44]. Regarding changes at 3-month follow-up, only the Dextrain group showed significant within-group change in MAL-AOU (Table 2), slightly below the ~ 1 point MAL change reported in the Excite-trial for patients receiving constraint-induced movement therapy [45]. Nonetheless, this 0.7 point change reflects a median change of ~ 14%, corresponding to minimal detectable change [36] suggesting that Dextrain training, in contrast to CT, led to meaningful and lasting changes in self-reported home use of the paretic hand.

### Enhanced recovery of dexterity components in the Dextrain group

Although we did not find significantly enhanced primary outcome after Dextrain therapy, our hypothesis that manual dexterity would be improved to a greater extent with Dextrain was largely confirmed by secondary outcomes, such as greater force tracking precision, better reaction times in independent finger movements and faster index-thumb finger tapping compared to the CT group, either immediately after training or at the 3-month follow-up, indicating sustained dexterity improvements (Table 2; Fig. 3). This suggests that selective training of finger movements and finger force promoted specific improvements. Of note, two of these measures were obtained in tasks that were specifically trained in the Dextrain, but not in the CT group. However, we also found a significantly greater improvement in maximal finger tapping speed at T1 (Table 2) which was not trained, showing transfer to non-trained dexterity components. Finger tapping speed is related to maximal finger motor output [25] and indicative of improved motor cortex function in post-stroke interventions [46], although we need to be cautious given that the improvement was not maintained at T2.

These results support the importance of intense finger movement training. The enhanced visual feedback of finger movements, providing continuous possibility of movement correction, together with the display of performance scores likely makes for more intense and more motivating finger training. Exercise intensity and motivation [46] are important factors for up-regulation of neural sensorimotor networks [47, 48], and could have contributed to less recovery in dropouts.

### Degree of initial motor impairment and recovery

Interestingly, the exploratory analysis revealed greater efficiency of Dextrain therapy in patients with most severe motor impairment, i.e. moderate weakness (Fig. 2C). Since maximal grip force is largely explained by degree of corticospinal tract (CST) lesion [8, 49] Dextrain therapy may enhance recovery of gross dexterity in patients with few residual CST connections. Perhaps Dextrain, through targeted finger movement training, permits more efficient plasticity of residual CST connections in these patients. The CST is essential for independence of finger movements [5, 7, 50] and training of individuated finger movements may thus help to up-regulate the residual CST connections. In turn, dexterity training may prevent adoption of less coordinated in-synergy finger movement strategies that likely rely more on reticulospinal tract connectivity [51]. Dextrain may thus present an opportunity for patients with moderate motor impairment to optimize residual CST connections, less achievable through conventional training. In comparison, patients with milder motor impairment improved similarly with Dextrain or CT, and maybe these patients had sufficient initial dexterity not inciting them to adopt in-synergy movement patterns.

### Enforced cortical inhibition associated with gross dexterity improvement

Despite the small sample, our findings suggest that, across both groups, stronger M1 inhibition after Dextrain or CT correlates with improved BBT performance. However, this was not the case for M1 excitability (resting motor threshold). SICI, which is reduced in the ipsilesional hemisphere after stroke [31, 52], showed a trend for improvement, and the greater the SICI increase the larger was the BBT improvement. This points to a role of cortical inhibition in recovery of dexterous hand use. SICI contributes to motor inhibition, i.e., the capacity to stop a planned or ongoing movement [28, 53, 54] and a recent study showed that a behavioral measure of motor inhibition, i.e. grip force release, can explain additional variance in BBT recovery not explained by other hand motor impairments [9]. Thus, improved BBT could partly be related to better ability to let go of grasped blocks, controlled through more rapid grip force release, in part mediated by stronger cortical inhibition. These findings suggest that finger and hand movement training may enhance cortical inhibition that translates to improved dexterous hand use. Since we did not measure SICI to other hand muscles, we cannot rule out reinforced surround inhibition after training [55].

### Study limitations

This study had limitations. First, the advantageous effects of Dextrain over CT in the non-parametric analysis on BBT changes at T2-T0, whether with raw BBT scores (Table 2), or with age- and gender-normalized BBT Z-scores (Additional file 2: Figure S2 and Table S1), was not confirmed in the linear mixed model analysis. The discrepancy between the non-parametric approach and the linear mixed models is likely due to reduced statistical power and less resistance of parametric approaches to outlier data. Nonetheless, the favorable effects of Dextrain over CT need to be confirmed in a larger sample.

Second, overall training dose consisted of only 12 1-h sessions. More sessions, e.g. ≥ 30 [40, 45], may have improved efficacy further. Yet, both treatment arms showed significant improvements. Enhancing initial training dose through tele-rehabilitation is warranted. Second, Dextrain therapy during the sub-acute (rather than chronic) phase may prove more efficient. This remains to be shown. Third, the patients included both patients with relatively recent and patients with old strokes. This may have impacted results, although we found no difference in time since stroke across groups (Table 1). Fourth, 1/3rd of patients were lost to follow-up, largely due to Covid-19 restrictions. Additional therapy after completed T2 could have improved attendance [56]. Fifth, the groups differed in age, potentially impacting results. However, results were similar when controlling for age, consistent with elderly and young subjects showing training-induced improvement in dexterity components [20]. Sixth, concerning choice of measurements, the BBT may have been influenced by impairments in proximal as well as distal motor control. While Dextrain focused on distal movements CT focused on both and this may have reduced the efficacy of the intervention therapy. We also did not use the Fugl-Meyer Assessment, the most commonly used upper limb motor impairment measure [57]. This measure was not included since the focus was on dexterity, however, it would have provided valuable complementary information on upper limb impairment. Finally, the use of subjective measures, such as the MAL, may have included a placebo effect which can be increased when using innovative technology [58].

## Conclusions

This study showed similar improvements in gross manual dexterity, in clinical sensory and motor impairment measures immediately after Dextrain training compared to conventional post-stroke training, with a strong trend for a greater effect of Dextrain therapy at the 3-month follow-up. Importantly, consistent with our hypothesis, Dextrain training enhanced recovery of several dexterity components, particularly in patients with moderate motor impairment. The Dextrain group also showed more favorable recovery in reported use of the paretic upper limb in activities of daily living at follow-up compared to CT group. Mechanistically, cortical inhibition increased in patients showing improved dexterous hand use. These findings are promising and larger multicenter trials using Dextrain, including more training sessions, are warranted.

## Supplementary Information

**Additional file 1.** CONSORT checklist.

**Additional file 2.** Supplementary Materials.

**Additional file 3.** Study data.

## Data Availability

The datasets used and/or analyzed during the current study are provided in Additional file 3 for the primary outcome. Other data are available from the corresponding author on reasonable request.
