# Supplementary material for: Efficacy of interactive manual dexterity training after stroke: a pilot single-blinded randomized controlled trial
Source: J Neuroeng Rehabil. 2023 Jul 18;20:93. doi: 10.1186/s12984-023-01213-9 (PMC10355015; doi:10.1186/s12984-023-01213-9)
Supplement: Supplementary file 2 — Additional file 2. Supplementary Materials. [file 12984_2023_1213_MOESM2_ESM.pdf]

[Tapez ici]

Supplementary Methods - Transcranial magnetic stimulation (TMS)

Motor evoked potentials (MEPs) were measured through electromyography recordings (EMGs) from first dorsal interosseous muscle (1DI) using surface electrodes (www.adinstruments.com). EMG signals were amplified with a CED 1902, and sampled at 1 kHz using a CED Power1401 under Spike2V6 (www.ced.co.uk). Transcranial Magnetic Stimulation (TMS) was applied over the ipsilesional motor cortical (M1) representation of the contralateral (right) 1DI through a figure-of-eight coil (7 cm diameter) connected to two synchronized Magstim 200 units (www.magstim.com). Optimal coil position was defined as the stimulation site inducing the largest 1DI motor evoked potentials (i.e., MEPs>50 mV) at the lowest intensity. A neuronavigation system was used during the entire session: coil position was maintained at target ±5 mm (and/or 5° shift) using default MRI scan (www.ant-neuro.com). SICI was measured as the % reduction of amplitude of the conditioned relative to the unconditioned motor evoked potential (MEP).

Supplementary Figure 1: tasks trained in the Dextrain sessions and effect of therapist.

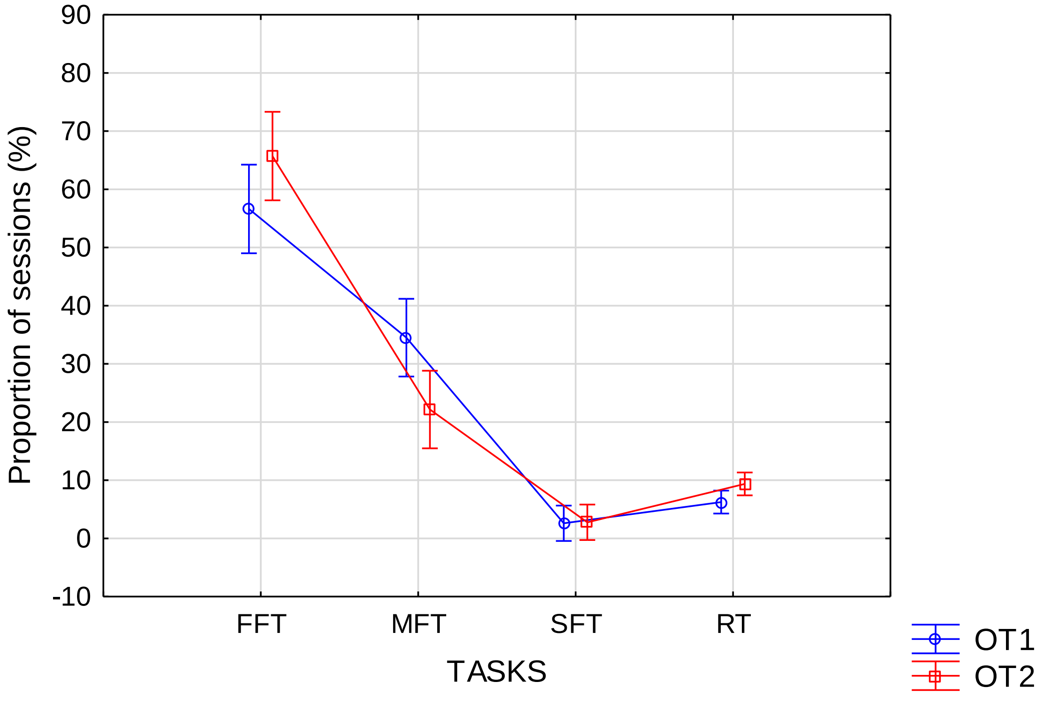

Dextrain tasks trained during 12 therapy sessions by occupational therapists (OT1 and OT2). Finger Force-tracking (FFT) was trained in about 60% of total tasks across the 4-week training period, followed by multi-finger tapping tasks (MFT), rhythmic tapping (RT) and sequence finger tapping (SFT). The SFT task was least used. ANOVA, including age as covariate, showed a similar choice of tasks by therapists (TASK*THERAPIST interaction, $F(3, 45)=2.46$, $P=0.08$).

[Tapez ici]

Supplementary Figure 2: median of BBT Z-score change (%, T2-T0) for Dextrain and CT group, respectively.

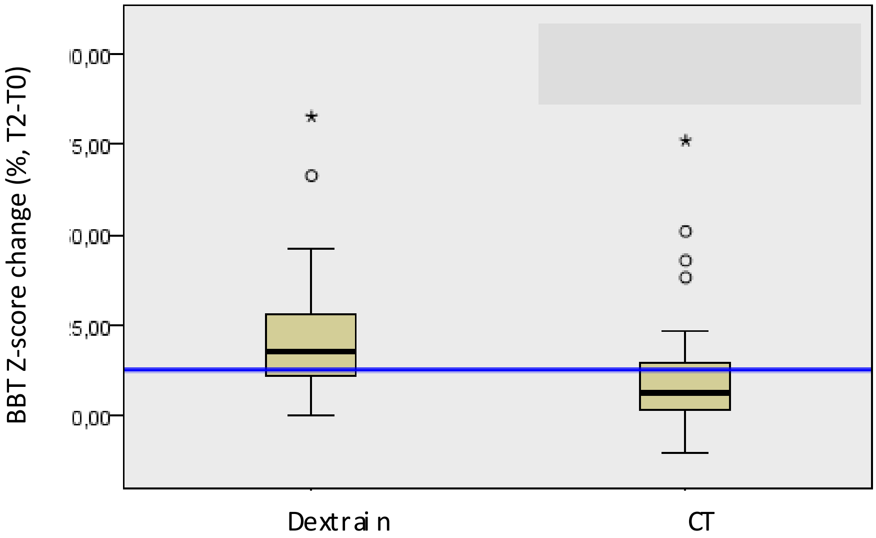

Median % change T2-T0 of normalized BBT in Dextrain and in CT group. Bold line=median, box=25%-75%. Asterisks and circles represent individual values: asterisks=extreme value, circle=outliers. Blue horizontal line: grand median=12.6. The age and gender normalized BBT scores were calculated according to Mathiowetz et al (1985). Missing data was imputed using the MissForest random forest imputation algorithm (Stekhoven and Bühlmann, 2012). Hodges-Lehmann estimator of median differences was used since data was skewed and non-normal: BBT Z-score change (%, T1-T0) Shapiro-Wilk W=0.82, P=0.0004, skewness=1.94 and BBT Z-score change (%, T2-T0) Shapiro-Wilk W=0.85, P=0.002, skewness=1.21.

[Tapez ici]

Supplementary Figure 3: dropout analysis: the number of dropouts at T2 was 8/21 in Dextrain group and 6/21 in CT group.

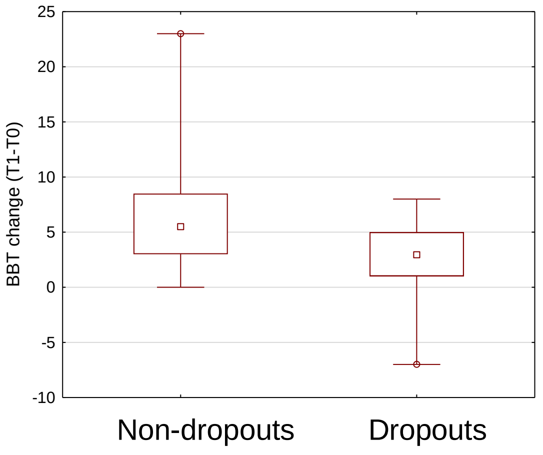

The training effect (whether CT or Dextrain) was significantly greater in the non-dropout patients compared to the dropouts (Mann-Whitney U Test, Z=2.36, P=0.018). Small squares depict median change directly after training, box shows interquartile range and whiskers the min/max values.

Supplementary Figure 4: correlation analysis between initial BBT scores (T0) and BBT change (T1-T0).

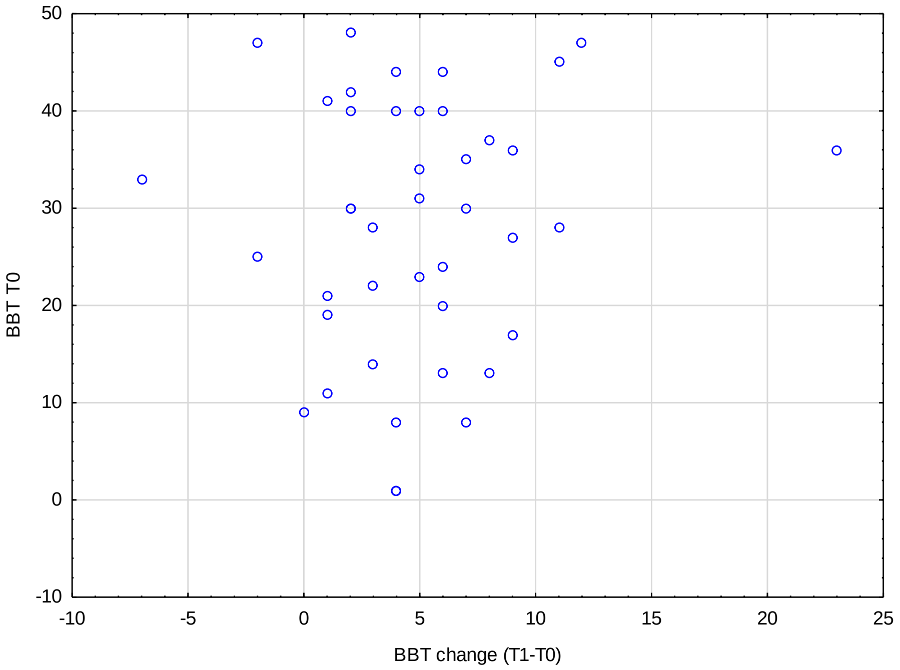

Spearman rank correlation test showed no association (N=42, Rho=0.08, P=0.63). Note the similar distribution of BBT change for subjects with initial BBT between 40-50 as for patients with lower initial BBT scores.

Supplementary Table 1: Hodges-Lehmann estimator on normalized BBT change Z-scores between CT and Dextrain groups, at T1-T0 and at T2-T0.

	median of differences [CL95%]	P-value (Mood median test)
Change (%) in normalized BBT Z-score (T1-T0)	2.3 [-4.8 to 8.1]	0.12
Change (%) in BBT Z-score (T2-T0)	9.4 [1.8 to 17.6]	0.031

Supplementary Table 2: linear mixed model results on primary outcome (BBT) at three time points (T0, T1 and T2) including age as fixed effect covariate.

Source	Numerator DoF	Denominator Dof	F	Sign.
**Constant**	1	40.16	8.63	0.005
**GROUP**	1	40.84	1.64	0.208
**TIME**	2	34.43	23.51	0.001
**GROUP*TIME**	2	34.43	1.32	0.281
**AGE**	1	39.3	0.183	0.671

Supplementary Table 3: linear mixed model results on age and gender corrected BBT performance at three time points (T0, T1 and T2). The BBT Z-scores corresponded to BBT scores normalized to age and gender norms (Mathiowetz et al., 1985). Missing data (from T2) were not replaced in this analysis.

Source	Numerator DoF	Denominator Dof	F	Sign.
**Constant**	1	40.07	223.69	0.001
**GROUP**	1	40.07	0.65	0.425
**TIME**	2	32.99	26.59	0.001
**GROUP*TIME**	2	32.99	1.20	0.313

Supplementary Table 4: linear mixed model results on age and gender corrected BBT performance at three time points (T0, T1 and T2) with missing data imputed using the MissForest random forest imputation algorithm (Stekhoven and Bühlmann, 2012)

Source	Numerator DoF	Denominator Dof	F	Sign.
**Constant**	1	40	230.69	0.001
**GROUP**	1	40	0.84	0.365
**TIME**	2	40	23.87	0.001
**GROUP*TIME**	2	40	1.25	0.297

Supplementary Table 5: baseline characteristics and training-induced improvements in T2 dropout vs non-dropouts.

	Mann-Whitney Z-value	P-value
Age	-0.15	0.88
Time since stroke	-0.32	0.75
BBT T0	0.32	0.75
MMSE T0	1.17	0.24
BBT change (T1-T0)	2.36	0.018
Max tapping rate change (T1-T0)	2.01	0.04

We studied the characteristics of dropouts at 3-month follow-up (T2) using Mann-Whitney U test. There was no difference in age, time since stroke, motor or cognitive impairment level at baseline between dropouts and non-dropouts. BBT change (T1-T0) was higher in non-dropouts (median, IQR: 5.5, 3-8.5) compared to dropouts (3, 1-5). Maximal tapping rate change also showed greater improvement after training in non-dropouts (mean ± SD: 7.9 ± 6.6) compared to dropouts (3.6 ± 4.7).

Supplemental references

Mathiowetz V, Volland G, Kashman N, Weber K. Adult norms for the Box and Block Test of manual dexterity. Am J Occup Ther. 1985 Jun;39(6):386-91.

Stekhoven DJ, Bühlmann P. MissForest--non-parametric missing value imputation for mixed-type data. Bioinformatics. 2012 Jan 1;28(1):112-8.
